# Gamma activation spread reflects disease activity in amyotrophic lateral sclerosis

**DOI:** 10.1016/j.clinph.2025.2110823

**Published:** 2025-06-29

**Authors:** Michael Trubshaw, Katie Yoganathan, Chetan Gohil, Charlotte J. Stagg, Anna C. Nobre, Kevin Talbot, Mark Woolrich, Alexander G. Thompson, Martin R. Turner

**Affiliations:** a Oxford Centre for Human Brain Activity, Wellcome Centre for Integrative Neuroimaging, University of Oxford, Oxford OX3 7JX, UK; b Department of Psychiatry, University of Oxford, Oxford OX3 7JX, UK; c Nuffield Department of Clinical Neurosciences, University of Oxford, Oxford OX3 9DU, UK

**Keywords:** MEG, Magnetoencephalography, ALS, Amyotrophic lateral sclerosis, Neuronal activity, Motor task

## Abstract

**Objective:**

A non-invasive measure of cerebral motor system dysfunction would be valuable as a biomarker in amyotrophic lateral sclerosis (ALS). Task-based magnetoencephalography (tMEG) measures the magnetic fields generated by cortical neuronal oscillatory activity during task performance. Gamma activations are periods of high-power and high-frequency cortical oscillations integral to motor control.

**Methods:**

tMEG was undertaken during 60 bilateral isometric hand grip exercises in ALS (n = 42) and compared with healthy controls (HC, n = 33). Gamma activation spread (GAS) was estimated by calculating the number of activated regions during each 100 ms time-bin and compared statistically between groups. Gamma activation patterns were visualised by plotting each participant’s brain activity separately as a 2-dimensional video.

**Results:**

There was no difference in grip strength between groups. GAS was greatly increased in the ALS group compared to HC (p < 0.001) and correlated positively with rate of ALSFRS-R progression (t = 1.35, p = 0.023) and a fine motor sub-score (t = -1.18, p = 0.047).

**Conclusions:**

ALS was associated with a marked increase in regional spread of gamma frequency activation, greater in those with higher disease progression rates.

**Significance:**

The regional spread of gamma activity may reflect disease activity in ALS, with potential application as an experimental medicine readout.

## 1 Introduction

Amyotrophic lateral sclerosis (ALS) is a progressive neurodegenerative disorder of the motor system and its wider cerebral connectome. The median survival is 30 months from symptom onset but with significant clinical heterogeneity (Es et al., 2017). There is no highly effective disease-modifying therapy. Biomarkers that reflect disease activity are needed as outcome measures to improve therapeutic trial design (Kiernan et al., 2021).

Magnetoencephalography (MEG) measures the magnetic fields generated by cortical neuronal oscillatory activity. Recordings during muscular contraction provide a highly temporally and spatially localised measure of brain activity responsible for motor control and planning (Trubshaw et al., 2024). Disruptions to beta and gamma frequency oscillations have been described in ALS (Bizovičar et al., 2014; Proudfoot et al., 2016; Trubshaw et al., 2024). Beta (13–30 Hz) event-related desynchronisations are widespread reductions in beta oscillatory power associated with planning and initiation of movement (Wiesman et al., 2020). Beta oscillations act as a ‘motor brake’ and desynchronisation of beta oscillations act as a ‘release’(Heinrichs-Graham and Wilson, 2015). Gamma frequency activations (30–48 Hz) are periods of high-frequency cortical oscillations integral to motor control (Wiesman et al., 2020). They have a particular role in top-down control of goal-directed actions (Wiesman et al., 2020). High-gamma frequency oscillations (52–80 Hz) show brief, highly localised, and temporally-constrained activations in contralateral M1, critical to movement execution (Wiesman et al., 2020).

We sought to characterise the topological spread of MEG activity during a motor task as a potential biomarker in ALS.

## 2 Method

Individuals referred to the Oxford Motor Neuron Disease Care and Research Centre and diagnosed with ALS (n = 42) were eligible for study. A healthy control group with no significant medical or family history was recruited (n = 33), which included spouses and friends of individuals in the ALS group. Groups were matched for age and sex. A single asymptomatic individual heterozygous for the *C9orf72* hexanucleotide repeat expansion (the commonest monogenetic cause of ALS) was also studied separately. A repeat study was undertaken in 6 ALS and the asymptomatic *C9orf72* heterozygote (mean follow-up 8.3 months). Individuals with ALS were stratified using the revised ALS Functional Rating Score (ALSFRS-R, with rate of disability progression calculated as 48-ALSFRS-R/time from symptom onset in months) (Cedarbaum et al., 1999) and the Edinburgh Cognitive & Behavioural ALS Screen (ECAS) (Niven et al., 2015). Written informed consent was obtained from all participants, and the study received approval from the National Research Ethics Service Committee (17/SC/0277).

Participants were presented with a total of 120 trials (epochs). Each trial lasted 6 s (−1s – 5 s) with 0 s defined as the trigger cue. At trial onset (–1s), vertical bars appeared on either side of a fixation cross. A trigger followed (time point 0 s), which consisted of two red lines, displayed horizontally on each bar (representing each hand). The height of the two red lines signalled the target force and persisted on screen for 3 s. Participants had to match and maintain the target gripper force (12 N) during the 3 s. They were instructed to release their bilateral grip when the red lines dropped to the bottom of both bars (time point 3 s). The trial ended at 5 s, and the next trial began after a pause of 2 s. Grip length, grip strength, reaction time, and accuracy were recorded and compared between groups using Welch’s two-sample *t*-test and corrected for multiple comparisons via Bonferroni correction.

MEG was acquired using a MEGIN Triux Neo system. Participants underwent a T1-weighted structural MRI within one month for co-registration. For 8 participants, MRI was not possible, so the MNI_152 standard brain was used for co-registration instead. A standard pre-processing pipeline using the Oxford Software Library (OSL) (Gohil et al., 2024) was employed for artifact removal, coregistration, and source localisation (see Supplementary Material for details).

Baseline-corrected power in the canonical frequency bands of beta (13–30 Hz), gamma (30–48 Hz), and high-gamma (52–80 Hz) were extracted using time–frequency representations from each of the 120, 6 s epochs. Activation time courses were calculated for each region and frequency (as per the Glasser52 parcellation (Kohl et al., 2023)), whereby each region was either ‘activated’ or ‘deactivated’ for each 100 ms time bin. Activation and de-activation cut-off values were set separately for each frequency and region by calculating two standard deviations of the power time course across participants in the HC group (see Supplementary Material for full description of the analysis steps). A single figure per participant representing gamma activation spread (GAS) during the tonic grip phase of the task was calculated by taking the mean number of gamma-activated regions between 1–3 s post trigger.

The mean number (across trials) of activated and deactivated regions were compared between ALS and HC in each canonical frequency band and time bin using General Linear Models (GLMs), including confound regressors for age, sex, and missing structural MRI. Further GLMs were constructed assessing the effect of GAS on clinical scores in the ALS group only, including disease burden (ALSFRS-R), disease activity (ALSFRS-R progression rate), serum neurofilament light chain, cognitive score (ECAS), and upper motor neuron score (UMN-score) (Menke et al., 2014). Significance (p < 0.05) was determined using non-parametric permutation testing which holds limited assumptions about the distribution of the data (see Supplementary Material). All P-values were reported after correction for multiple comparisons across regions, time points and frequency bands.

Videos of gamma and high-gamma activation over time were plotted for each participant and evaluated qualitatively by an operator (MT).

## 3 Results

Participant demographics and clinical features are shown in Table 1. There were no significant differences between ALS and HC in grip length, grip strength, reaction time, or accuracy (participants were only required to maintain a light grip strength of 12 N).

**Fig. 1:**
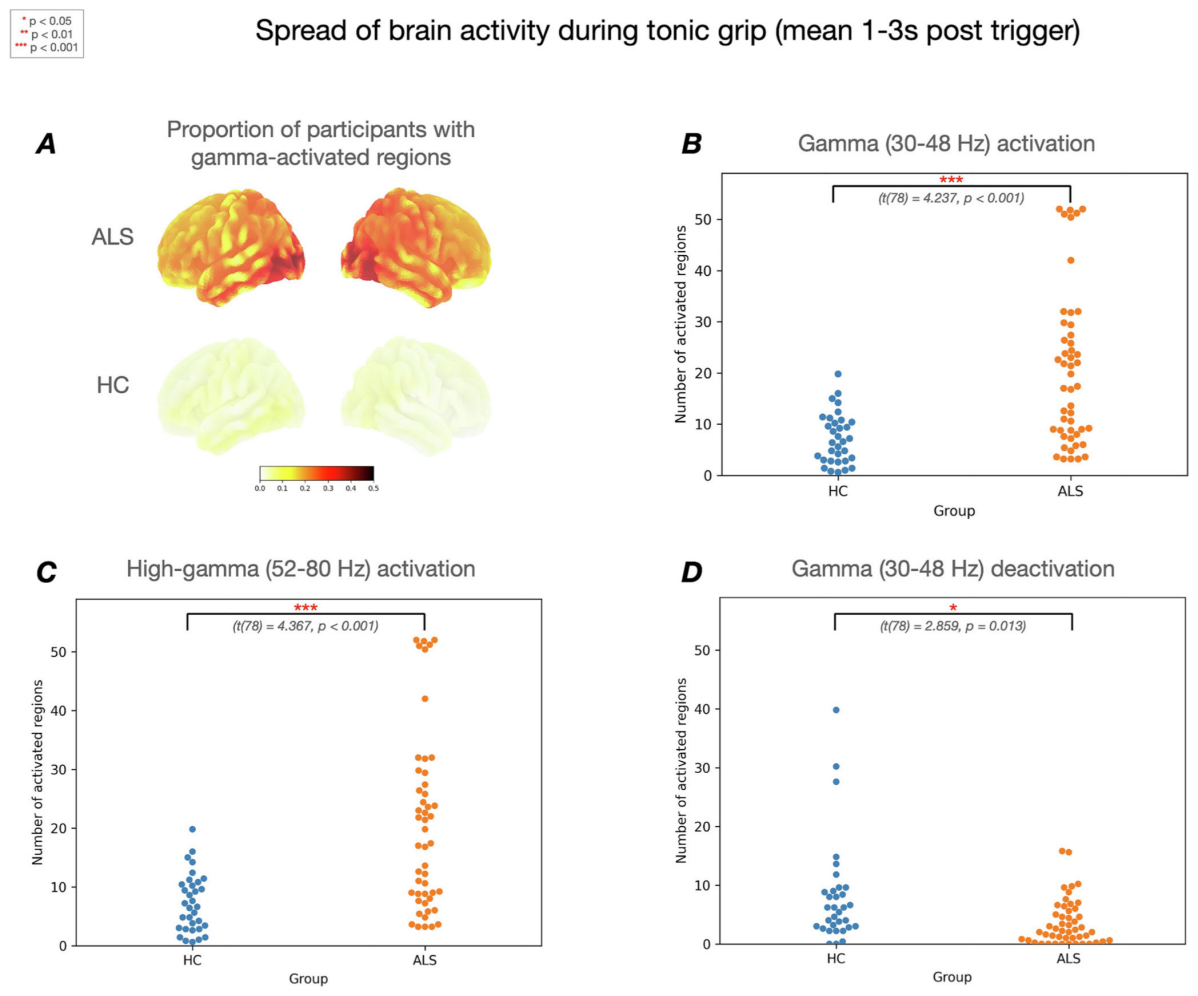
Brain activity spread during tonic grip. ***A*** shows regional gamma-activation during the tonic grip phase of the task (1–3 s post trigger) in the ALS and HC groups respectively. Darker regions represent a higher proportion of activation. ***B*** shows the significantly increased number of activated gamma regions in amyotrophic lateral sclerosis (ALS) compared to healthy controls (HC) in response to tonic grip (p < 0.001). ***C*** shows a significantly increased number of activated high-gamma regions in ALS vs HC in response to tonic grip (p < 0.001). ***D*** shows the significantly reduced number of deactivated regions in ALS compared to HC (p = 0.013).

The mean number of activated regions during the tonic grip phase of the task (1–3 s post trigger) was significantly increased in ALS versus HC in the gamma (20.30 vs 7.24, t(78) = 4.237, p < 0.001) (Fig. 1B) and high-gamma (33.89 vs 17.59, t(78) = 4.367, p < 0.001) frequency bands (Fig. 1C). The mean number of deactivated regions in the gamma band was also decreased in ALS (3.72 vs 8.12, t(78) = -2.859, p = 0.013) (Fig. 1D). There were no significant differences in beta activation or deactivation. Topographically, regional gamma activation in the ALS group was increased across the entire cortex, but was more heavily weighted to posterior cortical areas (Fig. 1A).

These effects were investigated further by plotting the number of activated and deactivated regions over time (Supplementary Fig. 2). This revealed that the highly significant increase in the number of activated regions in ALS in the gamma (Supplementary Fig. 2 – **middle row, right)** and high-gamma (Supplementary Fig. 2 **– bottom row, right**) bands occurred specifically during the tonic grip-phase of the task (see Supplementary Video – Slide 1 for spatial distribution of activations on a group level).

**Fig. 2:**
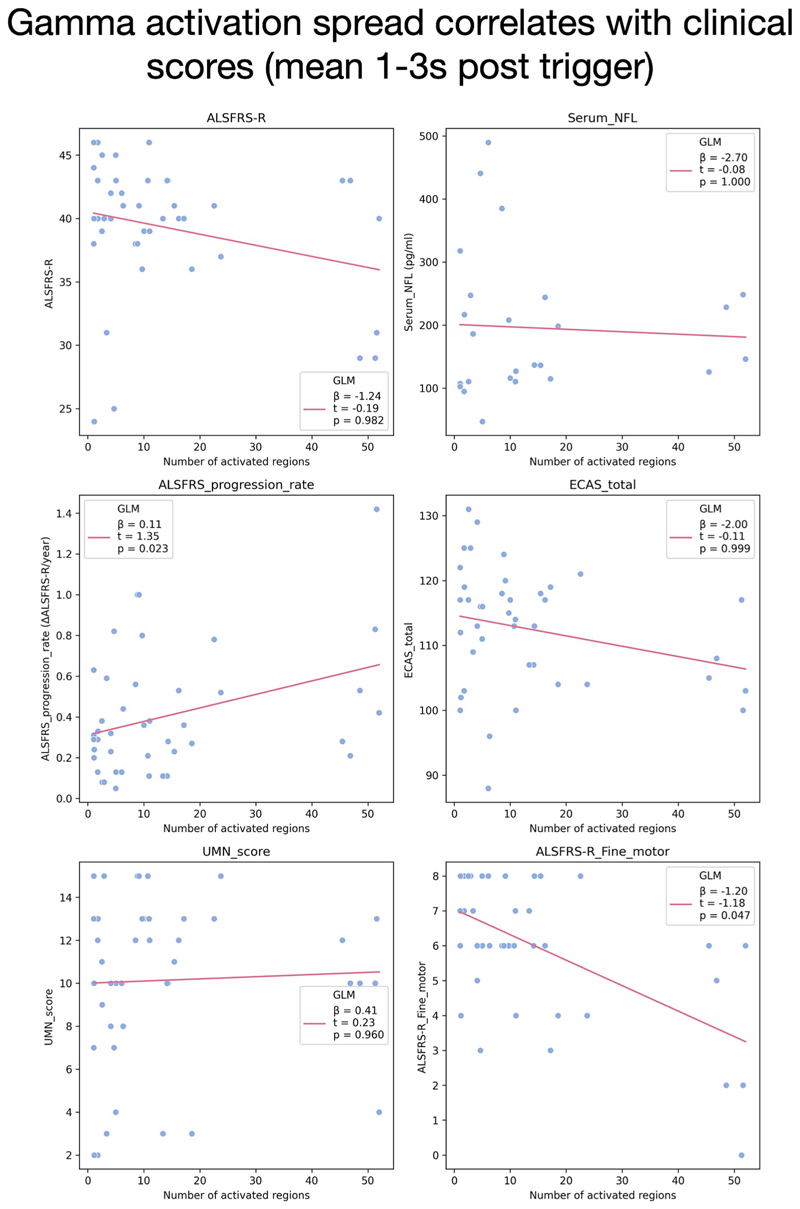
Gamma activation spread correlates with clinical scores. The mean number of gamma-activated regions between 1–3 s post-trigger (tonic grip phase of task) was correlated with clinical scores. Significant positive correlations were observed with ALSFRS progression rate (t = 1.35, p = 0.023) and negative correlations with ALSFRS fine motor subscore (t = -1.18, p = 0.047).

Increasing GAS was significantly positively associated with ALSFRS-R progression rate (β = 0.11, t = 1.35, p = 0.023) (Fig. 2 **– middle row, left**) but not with total ALSFRS-R score nor ECAS. A post hoc analysis exploring only the fine motor sub-score of the ALSFRS-R (based on handwriting and food handling scores) revealed a negative correlation (β = -1.20, t =-1.18, p = 0.047) (Fig. 2 **– bottom row, right**).

There was no significant change between scans (n = 6 ALS) in the mean number of activated gamma regions (12.83 vs 15.00, t = 0.349, p =1.000) or high-gamma regions (27.46 vs 28.23, t =0.098, p = 1.000). HC group gamma activations appeared qualitatively generally of low intensity, short-duration, and low number of regions in contrast to gamma in the ALS group, which was characterised by high-power, long-duration, and high number of regions activations, generally localised to the cortical regions expected to be associated with their functional deficits. The asymptomatic *C9orf72* heterozygote showed an increase in the mean number of activated gamma regions (4.2 in the gamma band and 12.2 in the high-gamma band) over 7 months (Supplementary Video 1).

## 4 Discussion

ALS is characterised by an abnormal spread of regional gamma activation during a motor task. Gamma activation spread (GAS) – the number of gamma-activated regions observed during the tonic phase of grip – was correlated with disability progression rate and fine motor subscores. This MEG study further supports an ‘interneuronal hypothesis’ of ALS pathogenesis (Turner and Kiernan, 2012). The “boundary shift” of positron emission tomography cortical activation during a motor task in ALS patients was the first to identify potentially defective inhibitory interneuronal local circuits (Kew et al., 1993), later supported histopathologically (Maekawa et al., 2004). It is notable that increased gamma power has been correlated negatively with GABA_A_ receptor density (Kujala et al., 2015), the latter having been shown to be reduced in ALS patients indirectly using flumazenil PET (Turner et al., 2005). Short interval paired transcranial magnetic stimulation studies have revealed a consistent cortical hyperexcitability in ALS (Menon et al., 2015; Yokota et al., 1996), that has been related to imbalance of inhibitory and excitatory local circuits (Van den Bos et al., 2018).

Generalised increases in gamma power in ALS have been described in both rest (Dash et al., 2024; Govaarts et al., 2022; Trubshaw et al., 2024) and task (Geronimo et al., 2016; Iyer et al., 2015). In this study, faster-progressing patients showed greater GAS. However, the relative stability in the 6 individuals studied longitudinally, suggests that it may represent disease activity rather than pathological load. The result in a single asymptomatic *C9orf72* heterozygote, at high risk of developing ALS, showed a deterioration in the appearance of their scan. Interpretation must be cautious, but the working hypothesis for a future dedicated study in a larger group is that GAS may be a pre-symptomatic marker of cortical adaptation or decompensation that precedes the phase of more rapid neuronal loss associated with a rise in neurofilament light chain (Benatar et al., 2018). More broadly, disruption to gamma and high-gamma suggests a failure of top-down cognitive control mechanisms integral to initiating movement (Wiesman et al., 2020). GAS correlated negatively with the fine motor disability sub-score and future work might usefully explore any link between GAS and biomarkers of lower motor neuron function such as CMAP amplitude and associated Neurophysiological Index.

The pathology of ALS, as with other neurodegenerative disorders, appears to involve marked changes in functional brain networks that have limited non-invasive tools capable of their measurement. The fact that GAS correlated positively with ALSFRS-R progression rate, but not with serum NfL implies that GAS may relate to aspects of functional network damage not detected by NfL but integral to disease progression. The scalability of MEG is currently a barrier to its use as an exploratory outcome measure in multi-centre therapeutic trials. However, with the advent of portable systems, these barriers should dissipate (Tierney et al., 2019). GAS measured through task MEG has significant potential in experimental medicine approaches to therapy development in ALS, where it could provide evidence of drug engagement with cortical network function. There is a particular value in exploring presymptomatic events through studies that include MEG in those at increased risk of ALS due to highly penetrant genetic variants. Delineating the compensatory and decompensatory phases of pathology in such individuals will be essential to realise the long-term aim of developing primary prevention strategies.

## Supplementary Material

Supplementary data to this article can be found online at https://doi.org/10.1016/j.clinph.2025.2110823.

Supplementary Material

Supplementary Video

## Figures and Tables

**Table 1:** Participant characteristics.

	HC mean (median)	HC Standard Deviation (interquartile range)	ALS mean (median)	ALS Standard Deviation (interquartile range)	Test Statistic	Corrected P-value
N	33	− -	42	− -	− -	− -
Age (years)	61.76 (66.00)	16.42	61.02 (63.00)	12.65 (15.75)	0.21	1.00
% Female	51.52	− -	33.33	− -	3.07	0.22
ALSFRS-R	− -	− -	39.29 (40.00)	5.27 (5.00)	− -	− -
ALSFRS-R Progression Rate (ALSFRS/month)	− -	− -	0.4 (0.32)	0.30 (0.32)	− -	− -
UMN Score	− -	− -	10.14 (10.50)	3.83 (4.75)	− -	− -
Disease duration (months)	− -	− -	29.31 (24.00)	20.97 (22.50)	− -	− -
ECAS score	118.78	9.64 (10.00)	112.56	9.22 (13.00)	2.63	0.03
	(119.00)		(114.00)			

## Data Availability

Data are not publicly available as they contain patient-sensitive information. Requests for access will be considered on submission to the authors. Sample code can be found at: https://github.com/mtrubshaw1/Trubshaw_2024_Gamma_Activation.
