# Supplementary material for: Gamma activation spread reflects disease activity in amyotrophic lateral sclerosis
Source: Clin Neurophysiol. Author manuscript; Available in PMC 2026 Feb 16. (PMC7618725; doi:10.1016/j.clinph.2025.2110823)
Supplement: Supplementary Video [file EMS212189-supplement-Supplementary_Video.docx]

**Supplementary material**

**Magnetoencephalography acquisition and analysis method**

MEG Acquisition

Prior to MEG acquisition, participant head shape was recorded using a Polhemus 3d tracking system, relative to three fiducial points on the nasion and preauricular landmarks. The location of five Head Position Indicator (HPI) coils, located on the participant’s nasion and bilateral supra-orbital and posterior auricular regions were continuously monitored in scanner space. The HPI coil and fiducial locations were digitised using the tracking system (Polhemus, EastTrach 3D) to define the subject-specific cartesian head co-ordinate system. Participants underwent a T1-weighted structural MRI within one month for MEG co-registration.

MEG processing

A standard pre-processing pipeline using the Oxford Software Library (OSL) (Gohil et al., 2024; Quinn et al., 2023) was employed for artifact removal, coregistration and source localisation (for full description of preprocessing steps on a similar dataset see Trubshaw et al. 2024) (Trubshaw et al., 2024). The standardised (z-transformed), epoched (split into trials) MEG time-courses with a sample frequency of 250 Hz were extracted. Epochs were extracted from -1s – 5s with 0s defined as the trigger cue.

The MNE-Python package was used to estimate a time-frequency representations (Morlet wavelet) of the MEG parcel time courses. Power in the beta (13-30 Hz), gamma (30-48 Hz) and high-gamma (52-80 Hz) frequency bands was calculated from the time-frequency transform of each epoch. These frequency bands were chosen due to their implication in movement (Cheyne, 2013).

Calculation of activation topographies

To allow for comparability between participants, MEG parcel time courses were baseline corrected by subtracting the mean power from 0s-0.6s (the rest period of the trial) for each frequency bin separately. The trial time course was then split into 100-sample time bins. For each participant and each time bin, the mean number of hyper- and hypo-activated regions were calculated across trials in each canonical frequency band separately. A region was counted as ‘activated' if its power exceeded a cut-off value placed above the baselined value of that region's power. Regions were counted as deactivated if they reached the same cut-off value below the baselined value. The cut-off value was set separately for each frequency bin and parcel by calculating two standard deviations of the power time course across participants in the HC group. A selection of cut-off values between 1 and 3 standard deviations were tested to ensure robustness of results. Results were also robust to the choice of sample time bin length between 25 and 250 samples.

Statistical analysis

The mean number of activated and deactivated regions ((de)activation spread) were compared between ALS and HC in each canonical frequency band and time bin using General Linear Models (GLMs) including confound regressors for age, sex, and missing structural MRI. The null hypothesis stated that there were no significant differences in the relevant metrics between groups. Statistical significance was determined using non-parametric permutation testing. Family-wise error rate correction for multiple comparisons, due to fitting a separate GLM to each frequency band and time bin was accounted for by using the maximum t-statistic method. T-statistics were calculated on the true dataset and then for 5,000 random permutations of that data. By taking the maximum t-statistic across all frequency bands and time bins in each permutation, a null distribution was created against which the t-statistic from the true dataset was compared. P-values were calculated by computing the proportion of maximum t-statistics from permutations that were smaller than the true t-statistic. Separate GLMs were constructed to assess the effect of gamma activation spread (GAS) on clinical metrics. Design matrices can be found in **Supplementary Figure 1**. P<0.05 was considered significant. All p-values are reported after correction for multiple comparisons.

**
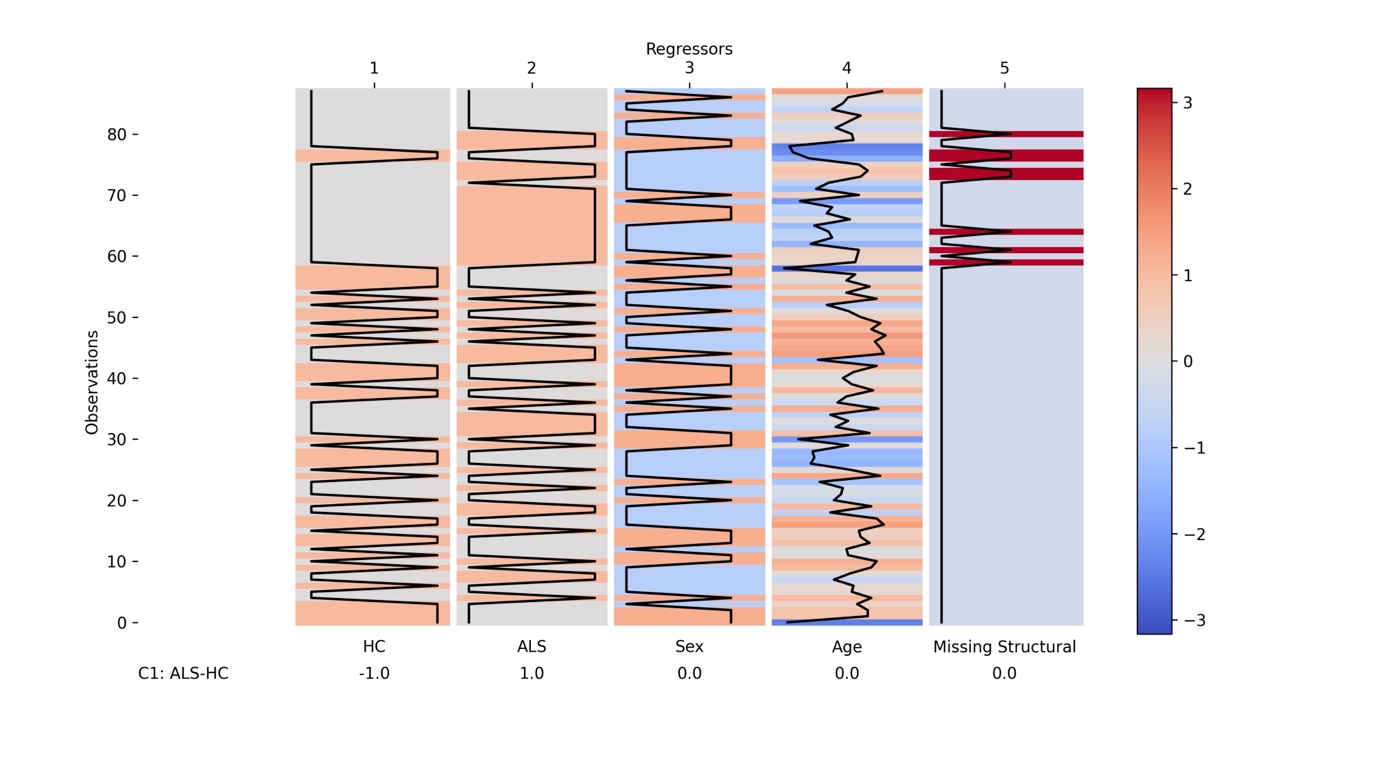
**

***A***

**
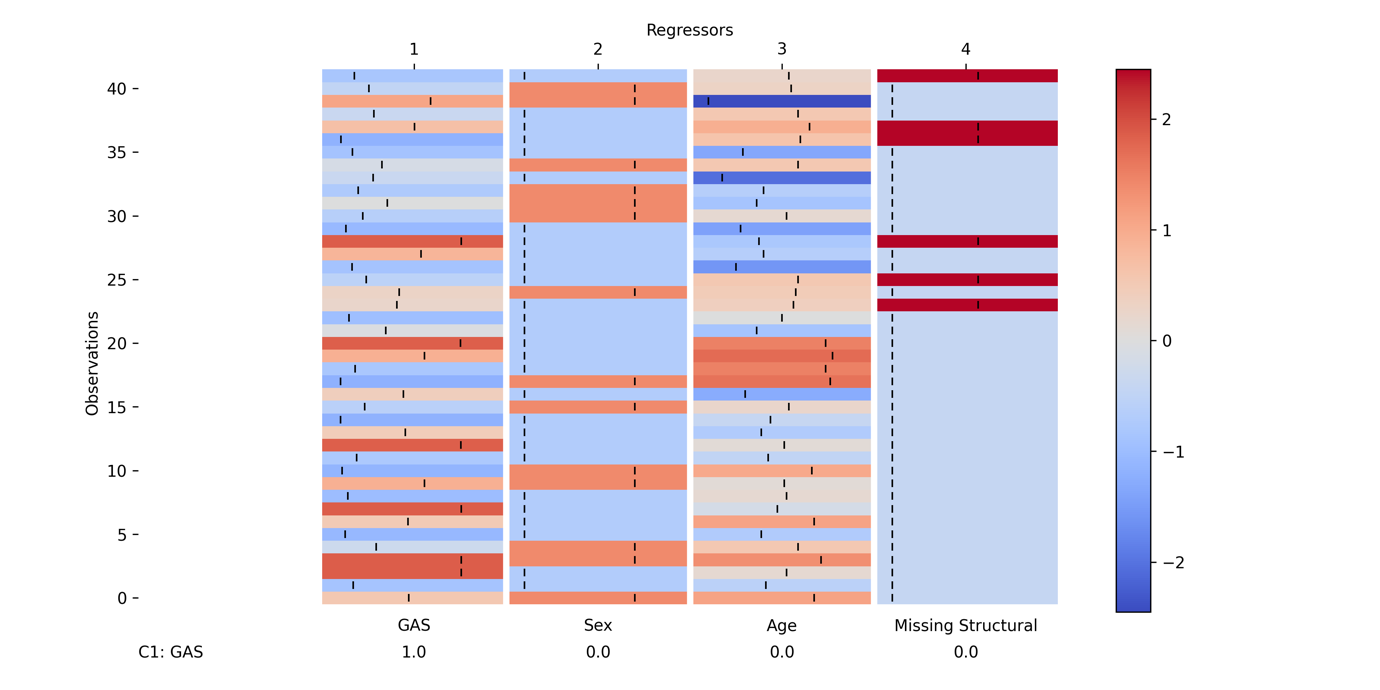
**

***B***

**Supplementary Figure 1 - General linear model design. *A* - group comparison**. Design matrix used to predict brain activity spread. The first regressor (HC) models the mean value of the metric across healthy controls (HC). The second regressor models the mean value of the network metric across amyotrophic lateral sclerosis (ALS) patients. The remaining regressors are included to model known sources of variability (age, sex, missing structural) across participants. This has the effect of minimising the impact of these confounds on the group means. The confound regressors are calculated by z-transforming the values for age, sex (1=female or 2=male) and missing structural (1 = not missing, 2 = missing) across participants. ***B* – Effect of gamma activation spread on clinical metrics.** The first regressor, gamma activation spread (GAS) models the clinical metric across participants according to the GAS. The remaining metrics model known sources of variability as in *A*.

**
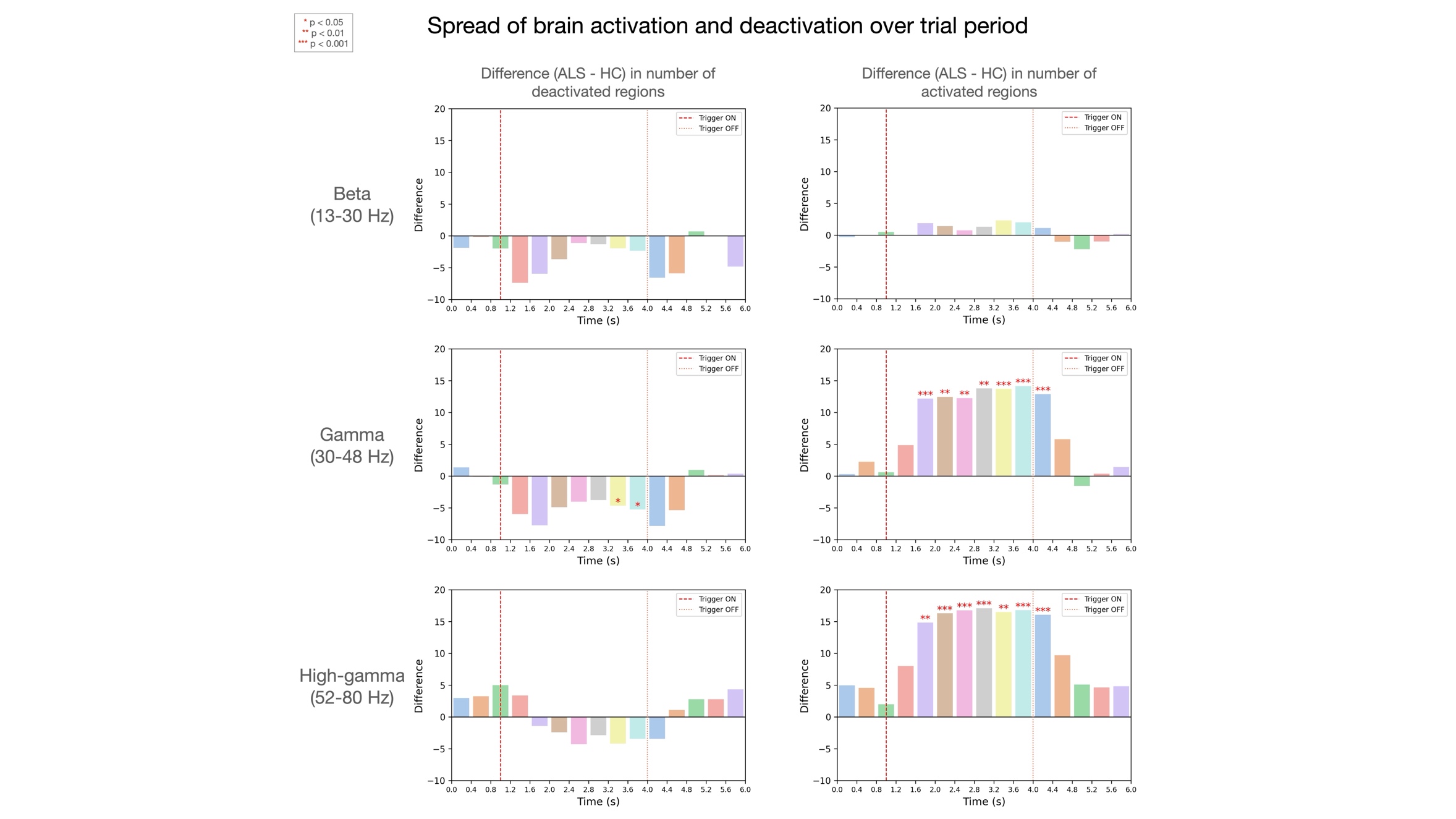
**

**Supplementary Figure 2 – brain activity spread over trial period**. The first column shows the number of deactivated, and the second the number of activated regions over the trial period. The first row shows the number of beta, the second gamma, and the third high-gamma deactivations and activations. In amyotrophic lateral sclerosis (ALS) vs healthy controls (HC), significantly increased number of gamma and high-gamma activations were observed between 1.6s and 4.4s. A significantly reduced number of deactivated gamma regions were observed between 3.2s and 4s.

**Supplementary Video 1**

Attached as supplementary file.**References**

Cheyne DO. MEG studies of sensorimotor rhythms: A review. Experimental Neurology 2013;245:27–39. https://doi.org/10.1016/j.expneurol.2012.08.030.

Gohil C, Huang R, Roberts E, van Es MW, Quinn AJ, Vidaurre D, et al. osl-dynamics, a toolbox for modeling fast dynamic brain activity. eLife 2024;12:RP91949. https://doi.org/10.7554/eLife.91949.

Quinn AJ, van Es MWJ, Gohil C, Woolrich MW. OHBA Software Library in Python (OSL) 2023. https://doi.org/10.5281/zenodo.6875060.

Trubshaw M, Gohil C, Yoganathan K, Kohl O, Edmond E, Proudfoot M, et al. The cortical neurophysiological signature of amyotrophic lateral sclerosis. Brain Communications 2024;6:fcae164. https://doi.org/10.1093/braincomms/fcae164.
